# Transcatheter Intracerebral Laser Photobiomodulation Therapy Improves Cerebral Blood Supply, Reduces Cerebral Atrophy and Reduces Dementia in Binswanger's Disease Long‐Term Results

**DOI:** 10.1002/alz70855_101584

**Published:** 2025-12-23

**Authors:** Ivan V Maksimovich

**Affiliations:** ^1^ Clinic of Cardiovascular Diseases named after Most Holy John Tobolsky, Moscow, Moscow, Russia

## Abstract

**Background:**

Improved cerebral blood supply causes a decrease in atrophic changes in the subcortical white matter of the brain, leads to decreased dementia severity and cognitive functions improvement in the treatment of neurodegenerative diseases such as BD.

The paper examines long‐term results (1‐10 years) after Transcatheter Intracerebral Laser Photobiomodulation Therapy in BD treatment.

**Method:**

The research included 18 patients aged 58‐81 (mean age 78), 11 (61.11%) men, 7 (38.89%) women, with BD of varying severity.

The examination included: CDR, MMSE assessment, cerebral MRI, MRA, CT, MSCTA, scintigraphy (SG), rheoencephalography (REG), cerebral multi‐gated angiography (MUGA).

According to dementia severity, patients were divided into mild stage CDR‐1 ‐ 12 patients, moderately severe stage CDR‐2 ‐ 6 patients.

All patients underwent Transcatheter Intracerebral Laser Photobiomodulation Therapy (PBMT).

**Result:**

According to SG and REG, only 1 year after PBMT, cerebral blood flow velocity and pulse blood filling improved in 18 (100%) cases. The obtained positive dynamics persisted throughout the observation period in all 18 (100%) cases.

According to CT and MRI, 1 year after PBMT, regardless of dementia stage, cerebral atrophic changes progressively decrease in 18 (100%) cases. In 2‐10 years, a further decrease in atrophic changes was detected in all 18 (100%) cases.

Clinically: 1‐2 years after PBMT, regardless of dementia stage, positive dynamics persisted, dementia signs decreased significantly, cognitive functions restored to 25‐29 MMSE points in 18 (100%) cases.

The obtained stable positive dynamics maintained throughout the ten‐year observation period in all 18 (100%) cases.

**Conclusion:**

Transcatheter Intracerebral Laser Photobiomodulation Therapy (PBMT) is a physiologically based and effective method of stimulating cerebral angiogenesis and neurogenesis in patients with BD. Complex intracerebral laser exposure causes cerebral collateral and capillary revascularization, improves tissue metabolism and causes the development of regenerative processes in cerebral tissues. Clinically, this leads to a decrease in dementia level and cognitive functions restoration. After the treatment, clinical effects last for a long time.

